# Structured learning for spatial information extraction from biomedical text: bacteria biotopes

**DOI:** 10.1186/s12859-015-0542-z

**Published:** 2015-04-25

**Authors:** Parisa Kordjamshidi, Dan Roth, Marie-Francine Moens

**Affiliations:** 1 KULeuven, Leuven, Belgium; 2 University of Illinois at Urbana-Champaign, Urbana-Champaign, USA

**Keywords:** Bacteria biotopes, Spatial information extraction, Biomedical text mining, Structured learning, BioNLP

## Abstract

**Background:**

We aim to automatically extract species names of bacteria and their locations from webpages. This task is important for exploiting the vast amount of biological knowledge which is expressed in diverse natural language texts and putting this knowledge in databases for easy access by biologists. The task is challenging and the previous results are far below an acceptable level of performance, particularly for extraction of localization relationships. Therefore, we aim to design a new system for such extractions, using the framework of structured machine learning techniques.

**Results:**

We design a new model for joint extraction of biomedical entities and the localization relationship. Our model is based on a *spatial role labeling (SpRL)* model designed for spatial understanding of unrestricted text. We extend SpRL to extract discourse level spatial relations in the biomedical domain and apply it on the BioNLP-ST 2013, BB-shared task. We highlight the main differences between general spatial language understanding and spatial information extraction from the scientific text which is the focus of this work. We exploit the text’s structure and discourse level global features. Our model and the designed features substantially improve on the previous systems, achieving an absolute improvement of approximately 57 percent over F1 measure of the best previous system for this task.

**Conclusions:**

Our experimental results indicate that a joint learning model over all entities and relationships in a document outperforms a model which extracts entities and relationships independently. Our global learning model significantly improves the state-of-the-art results on this task and has a high potential to be adopted in other natural language processing (NLP) tasks in the biomedical domain.

## Background

There is a rapidly increasing amount of literature available on the web related to biomedical information. Exploiting this literature is very difficult and time consuming for biologists. Automatic information extraction concerns extracting structured knowledge from diverse natural language texts and storing it in databases. This kind of extraction to make this information easily accessible to biologists, is increasingly seen as a necessity by the research community. Though this is a highly active research topic, the level of performance is still not satisfactory for many known tasks. The task we focus on in this paper is to extract information about bacteria and their locations from webpages. The locations indicate the places where given species live. Using such a system, biologists can easily query, for example, which bacteria live in the gut of a human; where Bifodobacterium Longum can live; or whether mammals provide a habitat for Bifidobacterium. The task is defined based on the Bacteria Biotopes (BB) [1] subtask of the BioNLP-ST 2013 shared task. BioNLP-ST 2013 is the third event in the BioNLP-ST series, which has attracted wide attention. The BB-task consists of three subtasks. Given a biological text, the first subtask is to detect habitat entities and classify them according to the categories specified in a habitat ontology. This ontology includes general categories like *human* down to very specific categories like *formula fed infants*. The second subtask is to extract two types of relations between given gold entities: Localization and PartOf relations. The given set of entities can be of type bacteria, habitats, or geographical locations. Localization relations occur between a bacterium and a habitat or geographical location, while PartOf relations occur between habitats. The third subtask is an extended combination of the other two subtasks: entities are detected in a text and relations between these entities are extracted. In this paper we focus on the third subtask, which is the most challenging one, and on which previous systems have performed relatively poorly.

The task of finding the location of biological entities is a kind of localization in the biomedical domain, so we aim to place it in the context of general domain-independent spatial language understanding, formulated in our previous research on the *Spatial Role Labeling (SpRL)* task [2-4]. SpRL considers generic location information expressed in free text about arbitrary entities– for example, finding the location of a book, when it is described in a sentence by referring to a table in a room. Here, we show the analogy between generic SpRL and the extraction of domain-specific localization relations in the biomedical literature. This analogy illustrates the challenges of applying generic NLP semantic extraction models to information extraction from the biomedical domain. The main contributions of this paper are as follows:

From the perspective of the BB-task:

1. We propose a scalable and generic machine learning model that jointly learns and predicts bacteria entities and their spatial relations. Here, we rely on a structured learning model that integrates expert knowledge on the possible relationships between biomedical entities and their constraints.
2. We substantially improve state-of-the-art results on the BioNLP-ST 2013 shared Bacteria Biotope task [5]; specifically, the F1 measure of our system is approximately 57*%* better than the previous results for localization relation extraction.
3. We elaborate on why joint and constrained machine learning has a high potential for many semantic extraction tasks involving biomedical texts.

From the perspective of the SpRL task:

1. We exploit the habitat ontology and the bacterium taxonomies for the extraction of spatial information: that is, we leverage external knowledge for the extraction of spatial roles and their relations.
2. We extend the SpRL to phrase level extractions from previous word level models, which is required to identify the correct bacterium and habitat entity mentions in biomedical texts.
3. We extend sentence level extraction to discourse (i.e. document) level extractions of entities and their relationships.

In the rest of this Background section, we first describe the problem that we tackle in this paper, and then explain the problem’s context from the perspectives of computational linguistics and spatial language understanding. At the end, we provide an overview of related research and previous results.

### Problem description

Our goal is the extraction of *bacteria* (specific biological entities) and their *habitats* (environments where a bacterium lives) from natural language text. For example, given a document *d* as below,



the task is to detect the biological **entities** of type E={Bacterium,Habitat}, and indicate which entities have **relationships** of type R={Localization}. We are given a training dataset in which these types of entities and relations are annotated manually and stored in annotation files with the following format,



The first column of this annotation contains the identifiers of annotated parts of a textual document, and the second column indicates the type of each annotated entity and relation. For this example, in the first two lines, two Bacteria entities have been annotated and identified by T1 and T2. In the case of entities, the third and fourth columns are the textual span of the annotated part of text and the next columns contain the actual text. Here, T1 identifies *Bifidobacterium longum NCC2705* which has the textual span from character 0 to character 30. In addition, two Habitat entities have been annotated and identified by T3 and T4. T3 identifies *human* which has the textual span from character 113 to 118 in the above document. The last line of annotation identifies a relation with identifier R1. In the case of relations, after indicating the type of relation in the second column (in this case, Localization), the types and identifiers of the entities linked by the relation are specified. In the example, the relation R1 holds between the entity T2 which is of type Bacterium and entity T3 which is labeled as type Localization (in the annotation files the term Localization has been used again instead of Habitat). The entity mentions can contain adjacent or nonadjacent words.

Given training data of this form, we aim to build a supervised learning model to predict such annotations given an input text. As noted before, this is the third subtask of the Bacteria Biotope (BB) shared task proposed in the framework of BioNLP-ST 2013.

**Figure 1:**
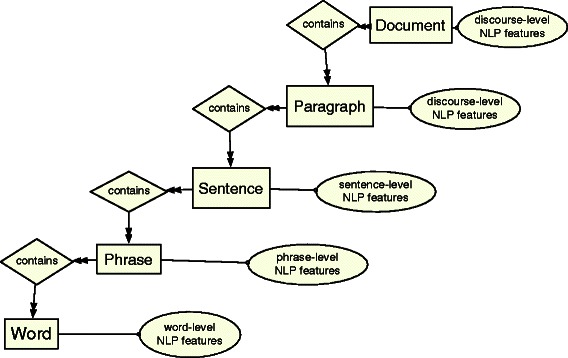
An input example structure represented as a document and its NLP features at different layers (document, paragraph, sentence,...) independent from the output representation and its elements.

Generally speaking, given an input document containing plain text, we read it into the linguistic structure represented in Figure 1. This structure has been shown in the form of an entity-relationship (ER) diagram. The rectangles show the linguistic units, called entities, and the diamonds show the relationships between these entities. Each input document *d*_*i*_ contains an arbitrary number of paragraphs *p*_*j*_ each of which contains a number of sentences *s*_*k*_. Sentences contain an arbitrary number of phrases *p* *h*_*l*_ each of which contains a number of words *w*_*m*_. Linguistic features are assigned to different types of input linguistic units (i.e. entities), and are noted as NLP features and shown in ovals. This input structure is independent of the elements we aim to predict in the output. We refer to the set of all input entities at various levels of granularity (i.e. document, sentence, etc.) as the *input space* and later we discuss the features that are used at each level.

**Figure 2:**
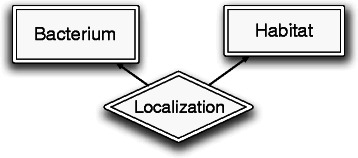
The output space of BB-localization task.

Figure 2 shows the output concepts to be predicted for this problem in terms of target entity and relationship types. Like the input structure, the output is represented by a simple ER diagram. This diagram shows that the output includes entities of two types (Bacterium and Habitat) and relationships of one type (Localization). In fact, each input linguistic entity of type Phrase is labeled as an output biological entity of type Bacterium or Habitat, or none of them. The pairs of phrases are labeled as having Localization relationship or not. We refer to the set of output variables containing all possible label assignments to phrases and their relations in a document as the *output space*. Finding the best assignments for an input document is the goal of our supervised learning model for this task. The formal specification of the problem and the input/output representation will be discussed later in the Methods section.

### Problem in the context of spatial language understanding

We view localization information extraction in the BB-task as a specific instance of general spatial language understanding. In this section, we introduce the spatial language understanding problem previously formulated as *Spatial role labeling (SpRL)* [2]. We discuss the new features of the SpRL model when it is applied to the biomedical domain.

#### Spatial role labeling

Domain-independent spatial language understanding is formulated as *spatial role labeling (SpRL)* in [2]. The SpRL model considers the extraction of a set of generic spatial roles and relations. This set includes, but is not limited to: the role of *trajector*, which is defined as an entity whose location or translocation is described in a sentence; the role of *landmark* which is defined as an entity by which we describe the location of the *trajector*; and the role of *spatial indicator* which is a linguistic signal that indicates the presence of a spatial relationship between *trajector*s and *landmark*s and provides information about the semantics of the relationship.

For example, in the sentence *“Give me the book on AI which is on the table behind you!”*, according to the SpRL scheme the word *book* is annotated as a *trajector* entity meaning that its location is described. The word *table* is annotated as a *landmark* entity meaning that it is the referring entity which describes the location of the book. The preposition *on* is annotated as a *spatial indicator* and triggers the spatial relationship between *book* and *table* and expresses the topological semantics of the spatial relationship. In SpRL, the *spatial relation*s are triplets containing the three types of entities mentioned above. For example, in the above sentence the triplet of (*on,book,table*) is annotated as a *spatial relation*. Moreover, the *table* has an additional role of *trajector* with respect to *you* (a *landmark*) and *behind* (another *spatial indicator*), composing the triplet (*behind,book,you*). In this specific sentence, these two triplets are annotated as *spatial relation*s and *table* is annotated twice with two different roles. In the SpRL scheme, the formal semantics of spatial relations are also annotated [4], but this lies outside the scope of the current work.

#### SpRL customized to BB-Localization

**Figure 3:**
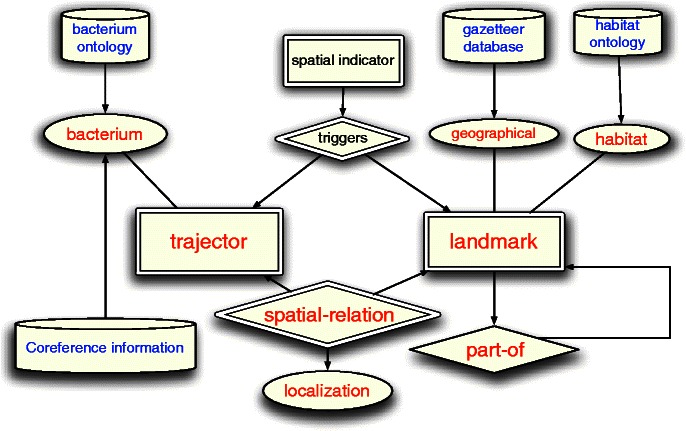
The output space when placing BB-localization in the SpRL framework. Double-line: the unknown elements relevant for our training/prediction model, Red: the output elements in the original BioNLP-task, Blue: external information that can be used, Black: output concepts of interest that are not annotated in the data.

By analogy to the general framework of *SpRL*, spatial roles and relations can be mapped to biological entities and localization relations in biomedical text. We consider the bacteria as a specific class of *trajector*s and habitats as a class of *landmark*s. Localization is a specific type of spatial relationship. Figure 3 shows how the BB-task is placed in the SpRL general framework. In this ER diagram, the double-lined shapes containing the red text are the corresponding SpRL elements that are targeted in the BB-task (*trajector*, *landmark* and *spatial relation*). In the BB-task, the pairs of entities have Localization relationships, in contrast to the SpRL in which the *spatial relationship*s apply to triplets. This difference is due to the absence of *spatial indicator* annotations in the BB-task data. As mentioned before, a *spatial indicator* is a signal that indicates the existence of spatial information, and a comparable concept – *trigger* – exists in various other biomedical event extraction tasks [6]. Similarly, the *trigger* is a part of text that indicates the occurrence of a specific relationship or event that relates entities in the text. Consequently, in the depicted diagram the *spatial indicator* box is not drawn as a direct part of the *spatial relationship*, but it is connected to the spatial entities with a dummy relationship called *triggers* to provide a full picture of the spatial roles and their correspondence to the BB-task entities.

We represent some more involved elements to clarify the context of the problem. The figure is augmented with the biomedical external resources depicted in cylinders. The ovals show the attributes of the spatial roles of *trajector* and *landmark*.

We observe a number of differences when moving from the general SpRL model to the domain-specific Bacteria Localization. Some conceptual differences originate from the basic difference between spatial language understanding – which tries to explore the semantics expressed in the language – and information extraction from scientific text – which tries to fill in databases about entities and their relationships – and which searches for linguistic evidence for that target. This basic difference leads to a different approach to annotating scientific text. For example, the Bacterium and Habitat are designated roles for the mentions and all are annotated, ignoring any Localization relationships stated in the text. More clearly, a bacterium is always a bacterium independent from the context, but in the general SpRL being a *trajector* depends on the spatial context, as it can be seen for the word *table* in our example.

Moreover, the entities’ roles in the BB-task are mutually exclusive, in contrast to the general SpRL. In other words, an entity which is a bacterium can not be a habitat, while in general spatial language an object can play the role of a *landmark* in one spatial relation while itself being a *trajector* in another spatial relation in the same sentence. In the example sentence from the previous subsection, the object *table* is annotated twice, once as a *trajector* and once as a *landmark*.

One can compare this to the following example in the BB-task data: *“This organism is found in adult humans and formula fed infants as a normal component of gut flora.”*. In this sentence, *gut* is a *landmark* (habitat) for the bacterium under discussion, but it is not a *trajector* for *formula fed infant*. Hence, in this domain a *landmark* can not be a *trajector*. The relationship between *gut* and *formula fed infant* is annotated as PartOf rather than Localization. A PartOf relationship can hold between two habitat entities (i.e. two *landmark*s). Note that the PartOf relationship in this biomedical context also has a spatial sense and can be considered as a type of spatial relationship; however we do not consider it as such in this paper.

From the linguistic syntactical and lexical point of view, in this domain, specific verbs often play the pivotal role of *spatial indicators*, as opposed to spatial prepositions, as in SpRL. Moreover, in the SpRL model the spatial implications are ignored and only the direct location information is considered. In biomedical text the localization relations are often implied from the semantics of the verbs that relate bacteria and habitats. In the previous annotated example, the phrase *colonize the human gastrointestinal tract* implies that the human is a habitat for the subject bacterium. In the sentence, *“This organism can infect humans and sheep...”*, the phrase *can infect* means that humans or sheep are a habitat for the bacterium.

Finally, our computational models for the BB-task recognize the full phrase or mention of an entity, where in our previous models for SpRL, a sentence is tokenized into words and the words are labeled.

One advantage of working on SpRL for scientific text is that there are often well-defined domain-specific ontologies which can be used. For instance, bacterium taxonomies and well-designed habitat ontologies can help the joint recognition of the spatial roles of *trajector* and *landmark* and their *spatial relation* in the BB-task.

For example, a soft or an exact match between a mention in the text and the classes in the ontologies can be used as a feature for the learning models. More specifically, in the above examples the terms *human* and *infant* exist in a habitat ontology called OntoBiotope [7].

### Related works

The BB-task along with the experimental dataset was first specified in the BioNLP-ST 2011 shared task [8]. Three systems were developed in 2011 and five systems for its extended version were proposed in the 2013 shared task [1]. In 2011, three systems participated in the task: UTurku [9], JAIST [10], and Bibliome [11]. UTurku was proposed as a generic system which uses a SVM multi-class classifier with the linear kernel. It made use of named entity recognition patterns and external resources for the BB model.

The second system was JAIST, specifically designed for the BB task. It uses CRFs for entity recognition and typing and classifiers for coreference resolution and event extraction. The third system was Bibliome, also specifically designed for this task. This system was rule-based, and exploited patterns and domain lexical resources.

The three systems used different resources for Bacterium name detection: which are the List of Prokaryotic Names with Standing in Nomenclature (LPNSN), names in the genomic BLAST page of NCBI and the NCBI Taxonomy, respectively. The Bibliome system was the winner for detecting the Bacterium names as well as for coreference resolution and event extraction. The important factor in their performance was exploiting the resources and ontologies. They found useful matching patterns for the detection of entities, types and events. Using their manually drawn patterns and rules performed better than other task participant systems, in which learning models apply more general features.

In the 2013 edition of this task, the event extraction was defined in a similar way but an extension to the 2011 edition considered biotope normalization using a large ontology of biotopes called OntoBiotope. The task was proposed as the three subtasks we referred to in previous sections. Five teams participated in these subtasks. In the first subtask all entities have to be predicted, even if they are not involved in any relation. The participating systems performed reasonably well. However, the difficulty in this task has been boundary detection. The participating systems obtained very low recall for the relation extraction even when the entities and their boundaries were given.

The difficulty of the relation extraction task was partly due to the high diversity of bacteria and locations. The large number of mentions of different bacteria and their localization in the same paragraph made it difficult to select the right links between them. The second difficulty has been the high frequency of the anaphoric expressions. This made the extraction of relations that cross sentence boundaries difficult. The results from the strict version of the third task were very poor, due to the challenges of the boundary detection and link extraction tasks. This task is our focus and we approach the challenge of relation extraction using joint learning and inference in the framework of structured output prediction. For subtask three, there were only two participant systems. One system was LIMSI [12] which uses a CRF for extraction of the entities and their boundaries. For relation extraction it relies on manual syntactical rules which fail to yield a reasonable accuracy. Another system is TEES [13] which provided better results compared to LIMSI. However, the results are still poor (see Section Results and discussion). TEES uses SVM classification in two steps: first for entity detection and classification and then another SVM layer for the relation extraction. In our experiments, their approach is compared with structured SVM and joint learning of both layers. None of the proposed systems perform joint learning and prediction of the entities and relations as we do in our work.

Joint learning models have become increasingly popular in various NLP tasks. Our experimental results indicate the advantage of joint training and prediction for this task, which is consistent with evidence from other NLP tasks [14-16]. The most recent work using structured learning and designing joint learning models in the biomedical domain are those of Riedel et al. [14] for event extraction in the BioNLP-ST 2011 Genia and BioNLP-ST 2011 Infectious Diseases tasks, which improved on the best participating models for these shared tasks. They use a dual decomposition approach to solve the underlying inference for structured learning and prediction, while we use an off-the-shelf optimization solver, specifying appropriate constraints. Another search-based structured learning approach applied on the same BioNLP-ST 2009 and BioNLP-ST 2011 shared tasks on event extraction appears in [17]. Their experimental results confirm the advantage of the learning joint models compared to independent models for event extraction. The above-mentioned works were related to the biomedical domain, but in other computational linguistic domains there are more recent efforts using joint learning and exploiting global features. One example of such an approach is [18], where the authors extract events, event triggers and their arguments. The events are the counterpart of relations here and arguments are the entities involved in the relations. They exploit the features between triggers and event arguments. This is similar to our effort in this work when exploiting between-relation features and using the similarity between entities involved in different relations. A more recent relevant work is [19] where they perform the standard entity-relationship task considering *people*, *location*, *organization* and a number of relations between these entities. They go beyond using pairs of relation labels and even exploit features linking three relation labels. In our task we only deal with one type of relation and two types of entities, hence exploiting the features between two relations makes more sense in our problem case. However, we believe there is still a significant room for improvement of our model, and adding more global features among relationships might be a useful solution.

A counterpart to our approach for structured learning would be probabilistic graphical models for extraction of the entities and relationships [20]. The disadvantage of these approaches is the comparatively high computational complexity for the probabilistic inference when considering global correlations, particularly when using global hard constraints.

The structured learning model that we design here is similar to the model that we have used on other datasets and tasks, particularly for extraction of general spatial relations from language and the type of spatial relationships in [21]. Our previous results on those different data have shown that our model outperforms local classifier models, particularly in relation extraction. In this work we have used the same framework to extract spatial relations in the biomedical domain. The results confirm the advantage of our model for entity-relation extraction in the biomedical domain.

## Methods

In this section, we first describe the machine learning framework, and the features it uses, and the way we model the problem to exploit the structure. We then describe the experimental methodology, data and setup which is employed for the evaluation of our designed models.

### Structured learning formulation

We formulate entity and relationship extraction in the framework of **structured output prediction** [22,23]. In this learning framework, given a set of *N* input-output pairs of training examples $E=\{(x^{i},y^{i})\in \mathcal {X}\times \mathcal {Y}: i=1.. N\}$, a function $g: \mathcal {X}\times \mathcal {Y}\rightarrow \mathbb {R}$ is learnt to assign a score to each input-output pair instead of a direct mapping from to  as compared to classical learning settings. In this way, the prediction is performed by maximizing *g* with parameter vector *W* over *y* for a given input *x*,

(1) $$  h(x;W)=\arg\max_{y\in{\mathcal{Y}}} g(x,y;W),  $$

where

(2) $$  g(x,y;W)=\langle W,f(x,y)\rangle,  $$

*g* is assumed to be a linear *discriminant* function over the joint features of the input and output *f*(*x*,*y*); *W* denotes a weight vector and 〈,〉 denotes a dot product between two vectors. The trained function *g* parametrized by *W* should be constraint to assign a larger score to the actual output *y* ^*i*^ than all other possible wrong *y*s for each input *x* ^*i*^ in the training data. Hence, the learning is formulated as minimizing the violations from this constraint. A more sophisticated technique is to consider the loss between a wrong *y* and the ground truth *y* ^*i*^ during the minimization (see Section Loss function). However, in the structured output problems, usually there is a large number of possible *y*s per *x* ^*i*^ that leads to a large number of learning constraints when searching for the optimum *W* based on the above idea. A solution to this problem is to consider only the most violated *y* for each *x*. In other words, at each training iteration the following inference is solved per training example,

(3) $$ l(W)=\sum\limits_{i=1}^{N} \max_{y\in\mathcal{Y}}(g(x^{i},y ;W) -g(x^{i},y^{i};W)+\Delta(y^{i},y)).  $$

The inner maximization is called loss-augmented inference and finds the most violating output (i.e. the output that is highly scored by the current model and meanwhile has a large loss) per training example. This is the crucial inference task that must be solved during training in most structured learning techniques, such as structured SVMs and structured perceptrons.

### Features

We use a collection of linguistic features, as well as some features from the supporting resources and ontologies provided by the task organizers, to be able to detect the entities, their types, and the relations linking them. Since an input instance is a document, the employed features are assigned to the document’s building blocks at various layers, that is *words, phrases, sentences and paragraphs*. In this way, the context of the entities and relationships is taken into account when classifying them via the structured model. Table 1 shows four classes of features that we use assigned to the input components of type *words*, *phrases*, *pairs of phrases* and *pairs of relations*. We briefly describe these features and the motivation behind using them.

**Table 1:** **Local and global features of various input components**

**Type**	**Feature name**	**Description**
Word features	Lexical-form	Word surface that appears in the text
	Bio-lemma	Word lemma using a lemmatizer for biomedical domain which uses additional lexical resources [24]
	POS-tag	Part of speech tag of a word to exploit the syntactical information for training
	Dprl	Dependency relation of a word to its syntactic head which gives clues to the semantic relationships
	Cocoa	Word tag using Cocoa - an external resource of biological concepts
	Capital	If a word starts with a capital letter
	Stop-word	If a word belongs to a list of stop words
Phrase features	Head-features	The features of the word which is the syntactic head of a phrase
	nHead-features	The features of other words contained in the phrase
	Lexical-surface	Concatenation of the lexical form of the words in the phrase
	Phrasal-POS	The phrasal part of speech tag: the parse tree tag of the common parent of the words in a phrase
	NCBI-sim	Comparing the phrase and the list of bacterium names in NCBI
	Ontobio-sim	Comparing the phrase and the habitat classes in OntoBiotope
Phrase-pair features	Same-par	If two phrases occur in same paragraph
	Same-sen	If two phrases occur in one sentence
	inTitle	If bacterium candidate occurs in the title
	Verb	The verb in between the two phrases- if in same sentence
	Preposition	The preposition in between the two phrases-if in same sentence
	Parse-Dis	The distance between the two phrases using the parse tree
	Parse-Path	The path between the two phrases using the parse tree
	Heads-Lem	The concatenation of the lemma of the heads
	Heads-POS	The concatenation of the POS-tag of the two heads
	Dep-Path	The dependency path between the two heads
Relation-pair features	Same-B	If two relations have exactly the same bacterium candidate
	Sim-BH	Similarity of two relations based on the similarity of their bacterium and habitat candidates

We use the three terms *local*, *relational*, and *contextual* respectively to refer to the features that are related to a single identified input component (i.e. an input entity or relationship), the features that are related to the context of an identified input component, and the features that are explicitly related to more than one identified input component. Relational features are sometimes referred to as global features in related works [18,19].

**Local and contextual features of words.** These features are used to help entity detection. The employed word level features are listed in Table 1 in the first set of rows and are described briefly.

The Cocoa feature in the above table is based on external information given by the task organizers. The Cocoa annotations map words to 37 predefined categories such as Cell, Organism, Body-part, Company, Food. These categories can clearly help us to recognize the entities in our interest.

**Local and contextual features of phrases.** As the entity labels are assigned to phrases rather than single words, we use more combinatorial features of phrases in our model based on the above-mentioned word-level features. The phrase-level features are listed in Table 1. To measure the similarity with NCBI and OntoBiotope, we use some binary features to represent the lexical overlap, containment and inverse containment between the phrases in the text and the ontologies. Before measuring the similarity, we remove some stop words from the habitat phrases and normalize the bacterium phrases by removing occurrences of {*s* *t* *r*.,*s* *t* *r*,*s* *p* *p*.,*s* *p* *p*,*s* *t* *r* *a* *i* *n*,*s* *p*.,*s* *p*,*s* *u* *b* *s* *p*} from the bacterium candidate phrases. The short explanations of the other features in Table 1 should be sufficient to reproduce them.

**Relational features of pairs of phrases.** We use a number of relational features between two phrases where one of them is a bacterium candidate and the other is a habitat candidate. These relational features are expected to help with recognizing which bacterium phrases are linked to which habitat phrases in the text. We assume that entity phrases in the same paragraph or in the same sentence are more likely to be linked to each other; hence we use the Same-par and Same-sen binary features. From looking at documents in the training/development data set, we observed that a bacterium name is often in the title of a paragraph and the whole text in that paragraph relates to that bacterium, so we use this as a binary feature (inTitle). Some relational features are only applicable for pairs that occur in the same sentence, such as Verb, Parse-Dis and Parse-Path. The Verb feature is the predicate (i.e. verb) in between the two phrase candidates for bacterium and habitat entities. As we pointed out in section SpRL customized to BB-Localization, the semantics of the verbs that connect two entities e.g. *colonize* or *populate* can imply the localization relationship between them; therefore we use this verb as a feature for detecting the relationship between two candidate entities. As sentences can be long and contain several verbs, we assume that the verb that is closest to a habitat candidate is the most informative.

Similarly, the Preposition feature is the preposition in between two candidate phrases and closest to the habitat candidate. Sometimes, the preposition is informative for recognizing the localization relation, for example in cases such as *in the human body* or *inside the liver*. Hence, we use both verbs and prepositions occurring in between two candidate entities as features.

We assume the entities that are related to each other should generally occur closer together in the parse tree, we therefore encode a feature (Parse-Dis) that reports the distance between the two phrases in the parse tree normalized by the number of nodes in the tree. Another relational feature (Parse-path) encodes our assumption that the syntactic path between two candidate phrases can help in recognizing whether they are semantically related to each other or not.

We assume the concurrence of two entities in the corpus can help distinguish the relationships between the entities in unseen documents, so we use Heads-Lem as a relational feature. Relying on the same idea, we use the relational features made by POS-tags of the phrasal head of the two entities i.e. Heads-Pos. Moreover, we assume when a specific dependency path is observed between two entities in the training data, this feature can help recognize the localization relation for the unobserved test examples, hence we exploit the Dep-Path as a feature too.

**Relational features of pairs of relations.** These are the most global type of features that we use in this work. We observe in the training data that when a bacterium is explained in a text, then most of the mentioned habitats in that text are related to the same bacterium. Hence, we took Same-B as a binary feature between two candidate relations. This feature gives a higher weight for instances of two relations with the same label when they have the same bacterium argument. Moreover, if a candidate bacterium and a candidate habitat have a localization relationship with each other, then the similar pairs also are likely to have the same relationship. However, the notion of similarity is challenging here.

For two bacteria we can only consider the lexical similarity as an indication that both of them refer to the same bacterium. Hence, we use the edit distance between the lexical form of the bacterium after normalizing their names, based on the rules described in the above paragraph about features of phrases.

For the habitats, measuring similarity can be more complex and related to their semantics. For each habitat candidate, we find the best matching node from the habitat ontology using the ontology terms themselves, and their *synonym terms* and *related terms* specified by the ontology. We then compare the best-matched ontology nodes and take this to be the similarity between the two habitat phrases. We measure this similarity using the edit distance *Edis* of the two strings which is normalized based on the length of the longest string and use the 1-*Edis* as the similarity measure.

Having the similarity of the arguments (i.e. Bacterium and Habitat) of two relations, now we are able to compare the two relations with each other. Two relations are similar when both of their arguments are similar. For this reason, we take the *geometrical mean* of the two computed similarities as the overall similarity of the two relations. If one of the similarities is low, this will have a sharper influence on the value of the geometrical mean, which makes it more sensible to use compared to other types of mean in our context. This similarity measure is a real-valued feature which is used for each pair of candidate relations.

Since the types of models that we use are robust and can deal with a large number of features, we made use of as many features as possible.

Our models weight the features that are more important according to the training data.

### Link-and-Label structured learning model

To specify the structured learning model, we use a representation called link-and-label (LAL) model [21,24].

In this model we use the notion of templates to represent the joint feature functions, output relations, correlations, and the constraints over the output variables imposed on the mentioned objective functions 2, 3. In the following sections we describe the LAL model and how the input and output of the structured learning model are represented in terms of labels and links for the BB-task.

#### Output representation

In the LAL model, the output variables are represented as a set of binary labels *l* whose size can vary per input example. In contrast to the varying size of the set of labels per input example, the type of the output variables in the model is a predefined fixed set. The labels which are related to the entities are called *single* labels. The labels which are related to the relationships are called *linked-labels*. The linked-labels can link not only the entities to each other but also to the relationships. In our problem setting the labels *l* can have the following types: *l*={*t* *r*,*l* *m*,*l* *o* *c*,*n* *r* *o* *l*,*n* *l* *o* *c*,*r* *r*}. The *tr* denotes a type of single label which indicates whether an input entity of type phrase is a bacterium, and *lm* denotes a type of single label that indicates whether a phrase is a habitat. In our model the linked-labels related to the localization establish a link between a pair of entities and we denote them by *loc*. We also consider the relation between pairs of relations and denote this type of linked-label by *rr*. The *rr* label indicates whether two given relations are both localization. The *nrol* label is an *auxiliary* single label type that indicates when a given entity is not a bacterium nor a habitat, and *nloc* is an auxiliary type of linked-label that indicates when the localization relationship between two arbitrary entities does not hold in a document.

#### Input representation

Each input instance in our learning model is a document. An input document is a set of input linguistic entities and relations according to the structure that we discussed in the Problem description section and is shown in the ER diagram of Figure 1. We represent these input as sets of input *candidate* entities that are relevant to each type of output label. In our problem setting, the sets of candidates for bacterium, habitat and non entities, that is, *tr*, *lm* and *nrol*, are selected from all phrases that belong to the input document (i.e. input phrases). These labels are only relevant for a subset of phrases (i.e. candidate phrases). In this work we define a phrase as a set of contiguous words that form part of one sentence (although in reality they are not necessarily contiguous).

Given each document, to generate the candidate phrases we use a chunker which is trained on the same training data to detect the boundaries of the phrases. The words which are not detected as a part of a mention by the chunker are removed, the phrases which have an overlapping token with the *OntoBiotope* [7] ontology of microbe habitats are used as habitat candidates, and the ones which have an overlap with the NCBI bacterium taxonomy database [25] are used as bacterium candidates. Any pair of bacteria and habitat candidates is taken as a candidate for the localization/nonlocalization relationship. Given the set of localization candidates, we generate an ordered list of them and take the pairs of *l* *o* *c*_*i*_ and *l* *o* *c*_*i*+1_ as the relation-relation, that is, *rr* candidates. We describe this choice in the experimental section.

We denote the candidates for the mentioned labels as *C*_*tr*_, *C*_*lm*_ and *C*_*nrol*_ respectively. The candidates for *nrol* actually belong to *C*_*tr*_∪*C*_*lm*_. The candidate sets for the localization and non localization relations i.e. *loc* and *nloc* linked-labels are equal, hence both are denoted as *C*_*loc*_. The candidate set for *rr* labels are denoted as *C*_*rr*_.

#### LAL objective function

In this section we expand the objective *g*=〈*W*,*f*(*x*,*y*)〉, and show its building blocks in our LAL model [21,24].

The model is specified with a number of templates $\{\mathcal {C}\}$. Each template $\mathcal {C}_{p}\in \mathcal {C}$ is defined with a type of label *l*_*p*_∈*l*, a local joint feature function *f*_*p*_(*x*_*k*_,*l*_*p*_), a candidate indicator function *C*_*p*_ and a block of the weight vector, *W*_*p*_. The global joint feature function is defined based on a number of local joint feature functions, *f*(*x*,*y*)={*f*_*p*_(*x*_*k*_,*l*_*p*_)}. These functions are the main components of the model templates. Each local joint feature function relates a part of the input *x*_*k*_ to a label in the output *l*_*p*_. In our model, we have: Bacterium template, Habitat template, Localization template, Localization-Localization template, and two more auxiliary templates called NonEntity template and NonLocalization template. The candidate indicator function indicates whether an input part is relevant to, and therefore should be combined with, the output label of each template. We use the same notation, *C*_*p*_, for candidate indicator functions and for their related set of candidates^a^.

The local joint feature function is computed as the scalar product of the input feature vector of *x*_*k*_, denoted by *ϕ*_*p*_(*x*_*k*_), and its output label $l_{p_{k}}$, that is $\phantom {\dot {i}\!}f_{p}(x_{k},l_{p})=\phi _{p}(x_{k})l_{{p}_{k}}$. This output label $\phantom {\dot {i}\!}l_{p_{k}}$ is the indicator function indicating label *l*_*p*_ for the component *x*_*k*_. The link-and-label (LAL) objective is written in terms of the instantiations of the templates and their related blocks of weights *W*_*p*_ in *W*=[*W*_*tr*_,*W*_*lm*_,...]. Using the actual type of the labels introduced in the Output representation section, *l*={*t* *r*,*l* *m*,*l* *o* *c*,*n* *r* *o* *l*,*n* *l* *o* *c*,*r* *r*}, we end up with the following objective after expanding the objective of equation 2:

(4) $$\begin{array}{*{20}l} &Obj=\sum\limits_{x_{k}\in C_{tr}} \langle W_{tr},\phi_{tr}(x_{k})\rangle tr_{k} + \end{array} $$

(5) $$\begin{array}{*{20}l} &\sum\limits_{x_{k}\in C_{lm}} \langle W_{lm},\phi_{lm}(x_{k})\rangle lm_{k}+ \end{array} $$

(6) $$\begin{array}{*{20}l} &\sum\limits_{x_{k}\in C_{nrol}} \langle W_{nrol},\phi_{tr}(x_{k})\rangle nrol_{k}+ \end{array} $$

(7) $$\begin{array}{*{20}l} &\sum\limits_{\langle x_{i},x_{j}\rangle\in C_{loc}} \langle W_{loc},\phi_{loc}(\langle x_{i},x_{j}\rangle)\rangle loc_{ij} + \end{array} $$

(8) $$\begin{array}{*{20}l} &\sum\limits_{\langle x_{i},x_{j}\rangle\in C_{nloc}} \langle W_{nloc},\phi_{nloc}(\langle x_{i},x_{j}\rangle)\rangle nloc_{ij}+ \end{array} $$

(9) $$\begin{array}{*{20}l} &\sum\limits_{\langle\langle x_{i},x_{j}\rangle,\langle x_{i^{\prime},}x_{j^{\prime}} \rangle\rangle\in C_{rr}} \langle W_{rr},\phi_{rr}(\langle \langle x_{i},x_{j}\rangle,\langle x_{i^{\prime},}x_{j^{\prime}}\rangle \rangle)\rangle rr_{iji^{\prime}j^{\prime}}, \end{array} $$

Given the weight vector *W*, the above objective is optimized during the prediction phase to provide the best assignments to the labels and link-labels for an input document. However, the output labels are not independent of each other and their value is constrained by the following constraints that originate from the definition of the labels of each template:

(10) $$\begin{array}{*{20}l} &\forall k,\ tr_{k}+lm_{k}+nrol_{k}=1 \end{array} $$

(11) $$\begin{array}{*{20}l} &\forall i,j, \ loc_{ij}+nloc_{ij}=1 \end{array} $$

(12) $$\begin{array}{*{20}l} &\forall i,j,\ tr_{i}\ge loc_{ij}, \ \ \ \ lm_{j}\ge loc_{ij}  \end{array} $$

(13) $$\begin{array}{*{20}l} &\forall i,j,i^{\prime},j^{\prime} \ loc_{ij}\ge rr_{iji^{\prime}j^{\prime}}, \ \ \ \ loc_{i^{\prime}j^{\prime}}\ge rr_{iji^{\prime}j^{\prime}}, \end{array} $$

Constraint 10 imposes the requirement that each candidate entity (i.e. phrase) is assigned only one label of *Bacterium*, *Habitat* or *none*. Constraint 11 imposes the requirement that each candidate relation is assigned the label *Localization* or not. The two constraints in 12, impose *Habitat* and *Bacterium* assignments when a *Localization* link is found. The integer variables refer to the binary labels here and the inference over this objective is solved using combinatorial constraint optimization solvers. Finally, when an *rr* joint label of two relations is active, it implies that both related localization relationships should be active; this constraint is formulated in the last two inequalities 13. These constraints impose the necessary structure on the output, and help make a joint prediction for a whole document compared to independently classifying the components of an input document.

To train the weight vector *W* we use the structured SVM (SSVM) model described in [22], which minimizes the general objective of 3. However, the SSVM technique treats the inner maximization, i.e. loss-augmented inference, of the objective 3 as a black box and does not provide a general solution to this. This loss-augmented inference objective is composed of the *g* function, which is expanded and explained in our LAL model case, and an additional loss component *Δ*. We describe the loss function of the LAL model and its solution to this inference in the following section to complete the specification of our training model based on SSVM.

#### Loss function

In the training process we iteratively perform inference to make a prediction that minimizes the errors made on the training set by our model by adjusting the weights of the features [22]. This is in fact minimizing a loss function. The loss function we use in the LAL training objective is defined as the weighted average of the loss of output components based on Hamming distance between the actual labels of candidates and their true label in the training data. For example, the loss of the model for all *tr* labels is computed as follows,

$$\Delta_{tr}={\frac{1}{|C_{tr}|}}\left(\sum\limits_{i\in C_{tr}}(1-2tr^{\prime}_{i})tr_{i}+\sum\limits_{i\in{C_{tr}}} tr^{\prime}_{i}\right), $$

where *t* *r* ^′^ is the ground-truth values of the *tr* labels and *C*_*tr*_ is, as before, the set of candidates for this type of label. Similarly, this loss is computed for other types of labels and averaged over all. This type of loss is easily decomposable in terms of output labels and linked-labels (i.e. *tr*, etc.). This is compatible with the way that the feature function is decomposed, hence the form of the objective function for inference during training is the same as the objective shown in equations 4-9, but with different coefficients.

In summary, we add the sum of losses of various labels to the objective and minimize the loss jointly. In this way, we perform structured loss minimization to train a model. However, we add the loss of the type of labels which are directly the target of the prediction, namely *tr,lm* and *loc*. This was an intuitive choice that led to improved performance in our experiments. Note that the above-mentioned structural constraints are imposed on the model during the training time while solving the loss-augmented inference. As the LAL objective of training and prediction time inference have a linear form, these are solved using off-the-shelf combinatorial optimization solvers.

### Experiments

In the experiments we aim to answer a number of research questions resulting from the previous efforts and systems designed for this task. In a review article on state-of-the-art systems participating in the BB-task [1], a number of challenges are mentioned. The most important challenge is performance in terms of F1. Particularly, the results on relation extraction have been very far from being useful in practical applications. The best proposed model for relation extraction yields only F1=0.14 [13]. This is analyzed as being due to the high frequency of anaphora and also the existence of many entity phrases in a paragraph which leads to the difficulty in finding the correct relations between entities. In this work we focus on improving relation extraction; we reach this goal by using rich contextual features and by jointly recognizing entities and their relations by means of the proposed structured learning model. The experimental research questions we aim to answer are the following:

**Q1.** Can joint entity/relationship recognition perform better than a model in which entities and the localization relationship are predicted independently?

**Q2.** Can joint entity/relationship training perform better than a model in which entities and localization relationship are trained independently?

**Q3.** Can we improve the state-of-the-art results on the BB-task, particularly with regard to relation extraction, by exploiting the joint learning framework?

#### Experimental setting

##### Data

We use the training, development and test data of the BioNLP-ST 2013 BB-task for experiments. The BB-dataset contains 105 documents: 52 for training, 26 for development and 27 for testing. There are 1347 bacteria species names, 1713 habitats and 1030 localization relations annotated in the training and development datasets.

##### Software tools and resources

We use the LBJChunker [26,27] to get the candidate phrases for the entities. The LBJChunker is trained with the training set of the BB-task. The linguistic features are extracted mostly based on the resources provided by the task organizers. The more complex combinatorial features such as the dependency paths, parse tree paths and others are constructed based on the provided parse trees and CoNLL format [28] data files of each document. A biolemmatizer is used [29] to add the lemma to the set of linguistic features. The NCBI taxonomy, OntoBiotope ontology and the Cocoa [30] external annotations are used for generating features as described in the Features section.

The Matlab interface of SVM-struct [31] is used for training. We have not performed any parameter tuning and used an initial setting for SVM-struct in all experiments. This setting is *c*=0.01, where *c* is the trade-off between training error and margin, and *e*=0.0001, where *e* is the error tolerance for the termination of training; we also used *o*=2, which means we made use of the margin rescaling option for rescaling our specified loss. The Matlab interface of the Gurobi [32] solver is used to solve the constraint optimization for training and prediction.

##### Evaluation

The evaluation metrics are *precision*, *recall* and *F1*. Precision is the proportion of the correctly predicted true labels to the total number of predicted true labels. Recall is the proportion of the correctly predicted true labels to the total number of actual true labels. F1 is the harmonic mean of precision and recall. We use our local evaluation system in our first set of experiments and then we use the standard online evaluation system provided by the task organizers. In our local evaluations, the training is performed using 52 training-set documents, and the evaluation is performed on 26 development-set documents. Our local evaluation is over the chunked development-set, meaning that the entities missed by the chunker are ignored. In other words, the reported recall will be bounded by the recall of the chunker, which is about 70*%* here. The goal of our local evaluation setting is to test our hypotheses about how various models should perform relative to one another. The final evaluation, on the other hand, is against the shared-task’s test data and reports the final performance and actual recall of our models to confirm our hypotheses and compare to the state-of-the-art models.

##### Models

We experimented with different settings based on how the various components of the objective function in lines 4-13 are considered together in finding assignments to the output variables. The various models are different in the level of globality/locality in the training and prediction time inference; we describe these settings in more detail along with the results in the following section.

## Results and discussion

The **first** experimental setting is called *learning only* (LO) [33]. In this model we train independent models for classification of the entities and for the classification of the relationships. We train the parameters of the first three components of the objective given in lines 4-6, which regard entity templates only, and we activate the constraint given in equation 10 to have a multi-class classification setting for recognizing the entities [34]. Training the weight vectors of the last two components of the objective function in equation 7-8, which are related to the relationship template, is performed independently from training the weights of the entity components. By activating the constraint given in equation 11 we have a binary classification of the pairs of entities to classify their relation as localization or not localization. After training the weights of entity and relation recognition, we can perform the prediction of the entities and relations independently considering the same objective components and same constraints. The results of this experiment are shown in Table 2, in the LO column. We use this as a baseline setting.

**Table 2:** **Local training/prediction vs. joint training and prediction over training/development sets; significant improvement made by the joint training models (IBT) on localization relationship (Loc) extraction**

		**LO**	**L+I**	**IBT-I**	**IBT+I**
Bac.	P	0.959	0.959	0.991	0.972
	R	0.993	0.994	0.970	0.978
	F	0.976	0.976	0.980	0.975
Hab.	P	0.977	0.977	0.987	0.977
	R	0.964	0.964	0.923	0.975
	F	0.971	0.971	0.954	0.976
Loc.	P	0.188	0.20	0.311	0.318
	R	0.274	0.268	0.584	0.580
	F	0.223	0.229	0.406	0.411

To answer the first research question **Q1**, in the **second** experiment we use the same trained model but perform joint inference for prediction of the entities and localization relationships. The joint inference is done using constraints defined in equations 10-12 when maximizing the objective function containing lines 4-8 for assigning optimum values to the output labels and links. The results are in the L+I column of Table 2. As can be observed, joint prediction slightly improves the recall of Bacterium (∼+0.001) but a significant improvement is made in the precision of the localizations (∼+0.12), yielding an improvement of about 0.07 in final F1 of localization. This means adding constraints that bind entities and relations to each other during prediction helps the precision of the relation extraction.

To answer the research question **Q2**, in the **third** and the **fourth** experimental settings we train the objective function including lines 4-8 by activating constraints 10-12. The two constraints in 12, bind the linked-labels of relationships to the single labels of entities. The results of the joint training model provide a great improvement on the extraction of the relationships in all the evaluation metrics, this is about 75 percent improve over F1 for the IBT-I model and about 80 percent improve over F1 for the IBT+I model. The difference between the last two models is that in IBT-I, we make a prediction independently although the training has been done jointly. In IBT+I, both training and prediction integrate a joint inference step. Overall the IBT+I is the best model, as expected.

The dramatic improvements by the joint learning and prediction models are made specifically on the relation extraction rather than on extraction of entities. In fact, extraction of the entities seems much easier than extraction of the relations in this task and the joint model stimulates the relation extraction when there is strong indication of the presence of both entities in a sentence or in a document (i.e. discourse). By using constraints during training in the IBT models, the parameter update is performed more conservatively as the incorrect predictions, still respect the structure of the output. In this way the unfeasible output predictions are not allowed to change the parameters of the model. This is a good and intuitive reason that explains why using constraints during training can yield better models.

In our final set of experiments and evaluation, to be able to compare our results with the state-of-the art models for this task, we train our models over the union of training and development sets and test on the standard test set composed of 27 documents whose annotations are not available. We evaluate our models using the online evaluation system of the BioNLP-ST 2013, shared BB-task [35] to be able to compare with the state-of-the-art models for this task. The description of the previous models, whose results are reported here, is provided in the Related works section. We also expand the global features of our IBT+I model and evaluate two more global models called IBT+IG1 and IBT+IG2. We describe the experimental settings of these two models later in this section.

These results on the test set with the standard task evaluation confirm the findings using training and development sets. We do not train our models for the PartOf relations and ignore them in the annotation files during training. The annotated data for the PartOf relation is very small and needs a different type of attention to deal with this problem. Hence, our models are trained and evaluated only for the Localization relations. However, we report the evaluation of our models when counting the missing PartOf relations as well (see results of IBT+1G1 (p)).

All variations of our IBT+I models strongly outperform the best shared task system TEES (SVM based model) [13], with about 0.081 improvement in F1-measure for the strict evaluation (considering missing PartOf) and about 0.09 improvement in F1 for the relaxed evaluation which ignores PartOf and does not require an exact match with the boundaries of the entities (see Table 3). LIMSI (CRF based model) is the other participant system and the last line of Table 3, IBT+I (2) shows the evaluation of the same IBT+I model but on the sentence level relations only. This evaluation ignores the missing relations that connect entities in different sentences. This result indicates our IBT+I model performs consistently well at the sentence and discourse levels. However, the recall of inter-sentence relations is lower as we do not explicitly deal with coreferences. Unfortunately, the sentence level evaluation of the BioNLP-ST 2013 participants is not available.

**Table 3:** **IBT+I vs. task-3 participants (TEES and LIMSI) evaluated on test set by the online system of the BioNLP-ST 2013 task; relations without gold entities; relaxed scores in parenthesis**

**System**	**P**	**R**	**F**
TEES	0.18 (0.61)	0.12 (0.41)	0.14 (0.49)
LIMSI	0.12 (0.15)	0.04 (0.08)	0.06 (0.09)
IBT+IG1 (p)	0.311	0.171	0.221
IBT+I	0.238 (0.596)	0.279 (0.561)	0.257 (0.578)
IBT+IG1	0.311 (0.594)	0.241 (0.483)	0.272 (0.533)
IBT+IG2	0.331 (0.588)	0.224 (0.431)	0.267 (0.498)
IBT+I (s)	0.241 (0.515)	0.436 (0.624)	0.311 (0.564)
IBT+IG1 (s)	0.305 (0.563)	0.400 (0.640)	0.346 (0.599)
IBT+IG2 (s)	0.327 (0.555)	0.367 (0.560)	0.346 (0.558)

(s) denotes the sentence level evaluation. (p) denotes that the strict evaluation also is punished by missing PartOf relations.

This experiment clearly provides a promising answer to the research question **Q3**, and confirms our above results and analysis based on the experiments that we did over training and development sets.

As mentioned earlier, with the IBT+IG1 and IBT+IG2 models, we exploit the potential of considering more global features in the structured output learning framework. We consider the additional terms in lines 9 in the main objective of the LAL model. These terms account for the global features between pairs of relations in the learning model. These types of features are described in the Features section. In this experimental setting, considering all possible pairs of relations through the whole document when solving the optimization leads to unmanageable memory requirements (the number of relation-relation pairs is *O*(*n* ^4^), where *n* is an estimate of the number of candidate entities for each role in the whole document). To alleviate this problem, we form a chain of all candidate relations and pair each relation only with its next relation in the chain. In this way each relation is paired with at least one other and the long distance dependencies between relations are considered indirectly for the sake of efficiency. Adding these terms to the objective implies considering additional constraints in equation 13, to impose the consistency between the *loc* labels that express the localization relationships and the new *rr* labels that express whether the localization relationships hold for two relations at the same time.

The experimental results are shown in Table 3. IBT+IG1 is the model that considers the Same-B feature and the IBG+IG2 considers both the Same-B and Sim-BH Relation-pair features described in Table 1. The strict localization results indicate a sharp improvement in the precision and a decrease in the recall, however the features have an overall positive impact as the F1-measure is increased by 0.01 for IBT+IG2 and slightly more for IBT+IG1 by 0.015. Though the IBT+IG2 model uses the more complex similarity measure compared to the binary exact-match in IBT+IG1, this was not very helpful for overall F1. Using both features yields an increase in the precision and a decrease in the recall. However, the overall result of the last two models shows that using the relational features between relations and the similarity between the entities involved in relations is a promising approach to improve the results. Particularly, this seems to be helpful for solving an important challenge of this task, namely coreference resolution. However, this challenge still exists, as using the usual similarity measures is not very helpful in this respect. Moreover, these similarity measures clearly can not deal with the anaphora resolution. For example, in the sentence mentioned in the Background section, the word *this* refers to the *Bifidobacterium*, hence the occurring habitats in that sentence are related to *Bifidobacterium* in the annotations. Recognizing this connection necessitates resolving the anaphora problem.

We believe the structured learning framework improves the relation extraction results because it provides the possibility of considering more global and structural features and it helps in dealing with numerous relation candidates where a large number of them are negative relations (i.e. selective parameter update). The large number of negative examples compared to the positive examples for the relations causes the binary classification of the relations to perform very poorly. In the joint learning setting we choose the best negative example per discourse jointly by doing inference and in this way we avoid the influence of the imbalanced data, which is a well-known phenomenon in relation extraction tasks. Our best model improves the best F1 measure of previous systems by 57*%*, ($\frac {22-14}{14}$), when considering the strict evaluation and taking missing PartOf relationships into account. This improvement is from *F*1=0.14 to *F*1=0.22. In future work we plan to build a model for jointly identifying anaphora/coreferences explicitly along with entities and relations. Another challenge for the future is the problem of nested entities. In the same mentioned example, both *human gastrointestinal tract* and its nesting entity *human* are annotated as habitats. We aim to use state-of-the-art research which considers the nesting problem in the chunking step to improve the results of the entity recognition [36,37]. By integrating these two extensions to our model we should have a relation extraction approach for biomedical texts which is suitable to be used in real world applications.

## Conclusions

Our investigation on the Bacteria-Biotope localization task (BB-task) illustrates the differences between spatial language understanding in general text and the extraction of spatial information from scientific text. These differences lead to different methods of annotating and variation in the background knowledge, constraints and the features that can be used in the two types of text. We designed a global structured prediction model for learning entities and the localization relationships in the framework of the BB-task. Our experimental results indicate a significant improvement resulting from the use of joint training and global features linking pairs of relations, when compared to training entity and relation extractors independently.

Our model significantly improves the state-of-the-art results on this task. There are a number of remaining challenges such as jointly resolving anaphora/coreferences, recognizing entities and their relationships, using more sophisticated similarity measures to compare relations, and dealing with nested entities, which could lead to further performance improvements for this task.

## Endnote

^a^ This notation style is commonly used in the literature for indicator functions.

## Abbreviations

SpRL: Spatial role labeling
SSVM: Structured support vector machines
SVM: Support vector machines
CRF: Conditional random fields
BB: Bacteria biotope
NLP: Natural language processing
POS: Part of speech
LO: Learning only
L+I: Learning plus inference
IBT: Inference based training
P: Precision
R: Recall
